# Supplementary material for: Association Between COVID-19 During Pregnancy and Preterm Birth by Trimester of Infection: Retrospective Cohort Study Using Large-Scale Social Media Data
Source: J Med Internet Res. 2025 Jul 9;27:e66097. doi: 10.2196/66097 (PMC12266298; doi:10.2196/66097)
Supplement: Multimedia Appendix 1 [file jmir-v27-e66097-s001.docx]

**Multimedia Appendix 1.** Preterm birth and term birth outcomes for maternal age-matched Twitter users with and without COVID-19 infection during pregnancy, based on manually validated self-reports in tweets.

| **Infected group (n=298)** | | | **Control group (n=298)** | | **Maternal age** |
| --- | --- | --- | --- | --- | --- |
| **Twitter user** | **Trimester of infection** | **Outcome** | **Twitter user** | **Outcome** |  |
| 4412508253 | Second | Term | 1003477874467266561 | Term | 18 |
| 2863325662 | Second | Term | 1013897013757382657 | Term | 19 |
| 718910074215862272 | Third | Term | 1009540265017856002 | Term | 19 |
| 1189688010490728448 | Second | Term | 1003981835855106048 | Term | 19 |
| 866388480938582016 | First | Term | 1062022321156882432 | Term | 20 |
| 1003283215698690049 | Third | Term | 1059620096921010176 | Term | 20 |
| 1444274174097334274 | Third | Term | 1048797016896229376 | Term | 20 |
| 940084926988173313 | Third | Term | 1036549131672674304 | Term | 21 |
| 247955785 | Third | Term | 1006688873882124288 | Preterm | 21 |
| 392091555 | Second | Term | 1011861958813941761 | Term | 21 |
| 2390042791 | Second | Term | 1004164089642270720 | Term | 21 |
| 2404861428 | Second | Term | 1003839791174217729 | Term | 21 |
| 2600476238 | First | Term | 1003504417621008384 | Term | 21 |
| 2650355485 | First | Term | 999438597689368578 | Term | 21 |
| 2859598378 | Second | Term | 1006325228388040704 | Term | 21 |
| 3357548013 | Third | Term | 983821855558549504 | Term | 21 |
| 4719365539 | First | Term | 976251119147118593 | Term | 21 |
| 754785911926198272 | First | Term | 974093015110291456 | Term | 21 |
| 993394273063260160 | Second | Term | 998386495927926785 | Term | 21 |
| 1078677151329718273 | Second | Preterm | 964682983910363136 | Term | 21 |
| 1163569922200559621 | First | Term | 962779274695577600 | Term | 21 |
| 1345936145746636800 | Third | Term | 957307132168343557 | Term | 21 |
| 1428121982776881161 | First | Term | 956738125056716801 | Preterm | 21 |
| 437952720 | Second | Term | 1019592547172343808 | Term | 22 |
| 471623424 | Second | Term | 965398738989658112 | Term | 22 |
| 865323703 | Third | Term | 953445517568172033 | Term | 22 |
| 2934351234 | Second | Term | 926260894920781824 | Term | 22 |
| 1037388142024646656 | Second | Term | 1028994652307943424 | Term | 22 |
| 1061967416001990656 | Second | Term | 1012590955164196864 | Term | 22 |
| 163738433 | Third | Term | 993568652174807043 | Term | 22 |
| 238034824 | Second | Term | 988554031130972161 | Term | 22 |
| 579505023 | Second | Term | 924700426230009860 | Term | 22 |
| 619740828 | Second | Term | 979420286893277184 | Term | 22 |
| 729573794 | Second | Term | 978362192625131521 | Term | 22 |
| 956315545 | First | Term | 966473986979835905 | Term | 22 |
| 1177799138 | Third | Preterm | 922991873911918595 | Term | 22 |
| 1490539958 | Second | Term | 957212813709201408 | Term | 22 |
| 2255394398 | Third | Preterm | 956411714391695360 | Term | 22 |
| 2326364562 | Third | Term | 944698554609754112 | Term | 22 |
| 2398025930 | Second | Term | 938910653334646785 | Term | 22 |
| 2444750641 | Second | Term | 909987642334371841 | Term | 22 |
| 2546189601 | First | Term | 926664559645085696 | Term | 22 |
| 2808753900 | First | Term | 925756908463517696 | Term | 22 |
| 2983462981 | Third | Term | 912886811390648320 | Term | 22 |
| 806645784720478208 | Second | Term | 912801765237682176 | Term | 22 |
| 968959435312791552 | Third | Term | 912749409468784640 | Term | 22 |
| 1071105151585435648 | Second | Term | 911257954686574593 | Term | 22 |
| 1092878497113088000 | First | Term | 900745249366003712 | Term | 22 |
| 1345184793437351938 | First | Term | 886408059966201857 | Term | 22 |
| 1438666084421361667 | First | Term | 904630963157835776 | Term | 22 |
| 283405162 | Second | Term | 922056248010334209 | Term | 23 |
| 601320687 | Second | Term | 953082125426798592 | Term | 23 |
| 1046480700 | Second | Term | 950759707857379328 | Term | 23 |
| 1561162171 | Third | Term | 950386413530505216 | Term | 23 |
| 3300518238 | Third | Term | 949713999112925185 | Term | 23 |
| 3309965665 | Third | Term | 947898563962527746 | Term | 23 |
| 258690273 | Second | Term | 946034199458467840 | Term | 23 |
| 340569939 | First | Term | 934962503410470912 | Term | 23 |
| 349799145 | Second | Term | 1030230523283529728 | Term | 23 |
| 534667979 | First | Term | 936394669256314880 | Term | 23 |
| 585059415 | Second | Term | 915466115442130944 | Term | 23 |
| 743214782 | Third | Term | 912071844903243776 | Term | 23 |
| 800610486 | First | Term | 910649553250000896 | Term | 23 |
| 879070386 | Second | Term | 894616879150530565 | Term | 23 |
| 1187426594 | Third | Term | 886636375923097600 | Term | 23 |
| 1347618794 | Second | Term | 883548575027916802 | Term | 23 |
| 1894209786 | First | Term | 876669379685081088 | Term | 23 |
| 2284352598 | Second | Term | 870351316471603200 | Term | 23 |
| 2398080485 | Third | Preterm | 865339177533153280 | Preterm | 23 |
| 3018430890 | Third | Term | 861449109642682368 | Term | 23 |
| 699042954241966082 | First | Term | 858935310243287040 | Term | 23 |
| 799788935723552768 | Third | Term | 857230673534496770 | Term | 23 |
| 969043756304551937 | Second | Term | 851749287629393921 | Term | 23 |
| 1092226688593780736 | First | Term | 846769513026736128 | Term | 23 |
| 1200511368551632898 | Second | Term | 886966838101508097 | Term | 23 |
| 1289287745563697155 | First | Term | 836797957940908033 | Term | 23 |
| 179712588 | Second | Term | 902303078287855616 | Term | 24 |
| 75837169 | Second | Term | 928093153806106625 | Term | 24 |
| 286346208 | Second | Term | 923562914224668673 | Term | 24 |
| 385109310 | Second | Term | 870076629048938496 | Term | 24 |
| 421798926 | Third | Term | 992976956395159553 | Term | 24 |
| 604852467 | First | Term | 963582602333405184 | Term | 24 |
| 618839862 | First | Term | 940025080641290241 | Term | 24 |
| 868881096 | Second | Term | 936041738606538752 | Term | 24 |
| 1368499267 | First | Term | 926153221781049345 | Term | 24 |
| 1381665535 | Second | Term | 923634424729698309 | Term | 24 |
| 1385801347 | Second | Term | 1018998638041853952 | Term | 24 |
| 1967955422 | First | Term | 921373036347043840 | Term | 24 |
| 2335831352 | Third | Preterm | 914659421678170112 | Term | 24 |
| 2367637525 | Second | Term | 913030106569994240 | Term | 24 |
| 2395666789 | Third | Term | 908191882655748096 | Term | 24 |
| 2558225062 | First | Term | 1012694772606267393 | Term | 24 |
| 2929564649 | First | Term | 881082920991166466 | Term | 24 |
| 4434222674 | First | Preterm | 945895501903392768 | Term | 24 |
| 701850836750770176 | Second | Term | 857750535402790912 | Term | 24 |
| 722865025346461697 | Third | Preterm | 850515229939290113 | Term | 24 |
| 1027928554640736256 | Third | Preterm | 841861871032492034 | Term | 24 |
| 1166377094584176640 | Second | Preterm | 838845779481509888 | Term | 24 |
| 1212918423300104193 | First | Preterm | 831540580874346496 | Term | 24 |
| 1308844543832449031 | Third | Term | 823780443917086720 | Term | 24 |
| 1316183081892614144 | Third | Term | 823696961136164866 | Term | 24 |
| 1399124534909886466 | Second | Term | 811785761217515520 | Term | 24 |
| 1438242794590642179 | Second | Term | 806014176598626304 | Term | 24 |
| 495053368 | Second | Term | 1017683743677566976 | Term | 25 |
| 4921128945 | Third | Term | 985485179136421888 | Term | 25 |
| 61825976 | Third | Term | 975511148878446592 | Term | 25 |
| 63112478 | First | Term | 948676800665800704 | Term | 25 |
| 66456914 | Third | Term | 942736246278848512 | Term | 25 |
| 134316211 | First | Preterm | 938154926299021322 | Term | 25 |
| 213706887 | First | Term | 937865389060644864 | Term | 25 |
| 243749111 | Third | Preterm | 917781610904383489 | Term | 25 |
| 317325381 | Second | Term | 823560089609076736 | Term | 25 |
| 378386974 | Second | Term | 904464059650772993 | Term | 25 |
| 412990195 | Third | Term | 892366114512130049 | Term | 25 |
| 548191451 | First | Term | 892135247751651329 | Term | 25 |
| 586768075 | First | Preterm | 885687203833294849 | Preterm | 25 |
| 586999578 | First | Term | 884656390421315584 | Term | 25 |
| 722256757 | First | Term | 866820614451077121 | Term | 25 |
| 920285570 | Third | Term | 863180731727532036 | Term | 25 |
| 2660018606 | Third | Term | 852283985342468096 | Term | 25 |
| 2709255689 | Second | Term | 846478034312069120 | Term | 25 |
| 751293679260700673 | Second | Term | 846368295641059328 | Term | 25 |
| 942147875546583040 | First | Term | 937369019056906242 | Term | 25 |
| 1251082595565285381 | First | Preterm | 837263737815977986 | Term | 25 |
| 1412873171208785921 | Third | Term | 831576226309480448 | Term | 25 |
| 1430005817764302855 | Third | Term | 866912539141189632 | Term | 25 |
| 54752383 | Third | Term | 932269457459896321 | Term | 26 |
| 386617584 | Third | Term | 939046159254818816 | Term | 26 |
| 388535972 | Second | Term | 900152768979116032 | Term | 26 |
| 1028106822798823424 | First | Term | 865723954195025925 | Term | 26 |
| 19215269 | Third | Term | 842184134407643136 | Term | 26 |
| 50532824 | Third | Term | 841813431460200448 | Term | 26 |
| 51368900 | First | Term | 836305703862169602 | Term | 26 |
| 166986949 | First | Term | 1064523805974945797 | Term | 26 |
| 170790485 | First | Term | 974044198344253441 | Term | 26 |
| 189997145 | Third | Term | 940762050007007232 | Preterm | 26 |
| 190363083 | Third | Preterm | 732723663221460993 | Term | 26 |
| 248863694 | Third | Term | 912383206221139968 | Term | 26 |
| 311840840 | Second | Term | 906226476911128576 | Term | 26 |
| 342543199 | First | Term | 871436170399555584 | Term | 26 |
| 393035530 | First | Term | 826751297932165120 | Term | 26 |
| 617158264 | Second | Term | 820508578436022272 | Term | 26 |
| 726628261 | Second | Term | 807685243650134016 | Term | 26 |
| 958832592 | Second | Term | 801590534406098944 | Term | 26 |
| 1152703484 | Third | Term | 787953559602094080 | Term | 26 |
| 1283630534 | First | Preterm | 785245249689890816 | Term | 26 |
| 804246919635734532 | Second | Term | 783002629433659392 | Term | 26 |
| 818223145085714440 | Second | Term | 781900352018718720 | Term | 26 |
| 926176140405985280 | First | Term | 764262232096145409 | Term | 26 |
| 1166173198670606336 | Second | Term | 748531914076790784 | Term | 26 |
| 1208119232761868291 | First | Term | 746317184826171397 | Term | 26 |
| 1214480827825786880 | Third | Term | 734155918451965952 | Term | 26 |
| 896529222 | Second | Term | 952964388817981440 | Term | 27 |
| 974067177396477952 | First | Term | 926504025763209216 | Term | 27 |
| 40600079 | Second | Term | 848543910855143425 | Term | 27 |
| 71216035 | Second | Term | 842077225738506240 | Term | 27 |
| 187416774 | First/Second | Term | 830379164767240192 | Term | 27 |
| 300593802 | First | Term | 824298193789784065 | Term | 27 |
| 322933713 | First | Term | 820780870923653120 | Term | 27 |
| 331715060 | First | Term | 818467410936266752 | Term | 27 |
| 426117875 | First | Term | 813665478199517185 | Term | 27 |
| 1174096790 | First | Term | 780946250803863552 | Preterm | 27 |
| 1220778205 | Third | Preterm | 759899930634887168 | Term | 27 |
| 2330948466 | Third | Term | 1019081809739444224 | Term | 27 |
| 2470264562 | Third | Term | 937439244561379328 | Term | 27 |
| 1018737940040646656 | Second | Term | 890283760792600576 | Term | 27 |
| 1087466145265774592 | Third | Term | 852279436460216320 | Term | 27 |
| 1101652846905380865 | Third | Term | 852169524375957509 | Term | 27 |
| 1192218976761401344 | Second | Preterm | 715381562234155009 | Term | 27 |
| 1313191898551640065 | Third | Term | 816356849515724800 | Term | 27 |
| 1353997839081246720 | Third | Preterm | 813387125043449856 | Term | 27 |
| 1481800765291343872 | First | Term | 809204693012541440 | Term | 27 |
| 899781833608638470 | Third | Term | 865664833064026112 | Term | 28 |
| 38102817 | First | Term | 795893308119625728 | Term | 28 |
| 43212944 | First | Term | 769836462598660096 | Term | 28 |
| 45744121 | Third | Term | 763516217638420480 | Term | 28 |
| 46277028 | First | Term | 742037566963261440 | Term | 28 |
| 50217544 | First | Term | 4221814918 | Term | 28 |
| 87543950 | Second | Term | 1002775980065222656 | Preterm | 28 |
| 150408100 | First | Term | 4019918236 | Term | 28 |
| 335920356 | Second | Term | 955601667105341441 | Term | 28 |
| 377792191 | Third | Term | 896044755938930688 | Term | 28 |
| 423624916 | Second | Term | 2902983178 | Term | 28 |
| 519265812 | First | Term | 838673121251102720 | Term | 28 |
| 1500447918 | Third | Term | 830868727423987714 | Term | 28 |
| 3087116074 | First | Term | 826286205280989184 | Term | 28 |
| 734056434652962816 | Third | Term | 3318128070 | Term | 28 |
| 997975534528290816 | Third | Term | 935741078006915073 | Term | 28 |
| 1052061171782770688 | Third | Term | 862953139976249344 | Term | 28 |
| 1086074569264455681 | Second | Term | 816835641539444736 | Term | 28 |
| 1191200763424448513 | Second | Term | 788136414919659520 | Term | 28 |
| 1195889803419619328 | First | Term | 3194924767 | Term | 28 |
| 1245799892372594689 | Third | Term | 715573860708397057 | Term | 28 |
| 1305851140358254594 | First | Term | 711004123035004928 | Term | 28 |
| 164102389 | Second | Term | 765372228640866304 | Term | 29 |
| 29745482 | Second | Term | 849083155994882048 | Term | 29 |
| 39058295 | First | Term | 1004024844189483008 | Term | 29 |
| 186887963 | First | Term | 705250346743042048 | Term | 29 |
| 280212508 | First | Term | 912006111800303621 | Term | 29 |
| 419291448 | First | Term | 909885800577425408 | Term | 29 |
| 483763566 | Third | Term | 903770059759394817 | Term | 29 |
| 498259824 | Second | Term | 824434595462418433 | Term | 29 |
| 741657187 | First | Term | 806525734168752128 | Term | 29 |
| 976355833 | Second | Term | 793514332533514240 | Term | 29 |
| 2170631820 | First | Term | 776080665712422912 | Term | 29 |
| 3239320980 | Third | Term | 760423825855119361 | Term | 29 |
| 743535950790336514 | Second | Term | 755558553750937600 | Term | 29 |
| 775703572948262912 | First | Term | 720346441760157696 | Term | 29 |
| 912106548989640704 | First | Term | 715186532857081856 | Term | 29 |
| 1023746007090307073 | Second | Preterm | 997180182418124801 | Term | 29 |
| 1129918413252505606 | First | Term | 956922510632800257 | Term | 29 |
| 1243262599099084800 | Second | Term | 924173152053231616 | Term | 29 |
| 1415496137306411008 | Second | Term | 887529323565113344 | Term | 29 |
| 18786857 | Second | Term | 996403746291662848 | Term | 30 |
| 20610400 | Third | Term | 4623765700 | Term | 30 |
| 20739682 | Second | Term | 971664718946603010 | Term | 30 |
| 38986616 | Third | Term | 3736227019 | Term | 30 |
| 153067265 | Third | Preterm | 888099025181921280 | Term | 30 |
| 260021732 | First | Term | 960809908445773824 | Term | 30 |
| 265756927 | Third | Term | 897731257051828226 | Term | 30 |
| 603085474 | First | Term | 871479028041035780 | Term | 30 |
| 2369673072 | Third | Term | 849499366134091776 | Term | 30 |
| 2372356009 | First | Term | 4149820109 | Term | 30 |
| 2502792794 | First | Term | 806561840079704065 | Preterm | 30 |
| 4740780290 | Second | Term | 805131376248766464 | Term | 30 |
| 807664262290124800 | Second | Term | 743558856840667136 | Term | 30 |
| 1055161748905312257 | First | Term | 728089445564456960 | Term | 30 |
| 1126485138639073282 | Third | Term | 723232281809162241 | Term | 30 |
| 1245878339266625537 | Third | Term | 706668459355025408 | Term | 30 |
| 1294457260702236673 | First | Term | 3259124324 | Term | 30 |
| 1306231011408318465 | First | Term | 700896901433061376 | Term | 30 |
| 1362531448562057216 | Second | Term | 4716341008 | Term | 30 |
| 16939745 | Third | Term | 3394951679 | Term | 31 |
| 55479164 | Third | Term | 795662396102676482 | Term | 31 |
| 95211720 | Second | Preterm | 840987497169604609 | Term | 31 |
| 24361878 | Third | Term | 766606144467075072 | Term | 31 |
| 25757522 | Second | Term | 924377023392505857 | Term | 31 |
| 46562179 | First | Term | 941975588255956992 | Term | 31 |
| 75834153 | Third | Term | 3154558324 | Term | 31 |
| 129535455 | Third | Term | 859977217400594433 | Term | 31 |
| 334268191 | Third | Term | 785221407802294272 | Term | 31 |
| 385411143 | First | Term | 783377427166818304 | Term | 31 |
| 957892232 | Third | Term | 754421112772169728 | Term | 31 |
| 1326064430 | First | Preterm | 713634873647435776 | Term | 31 |
| 1206627802038050816 | Second | Term | 701381903279067136 | Term | 31 |
| 1263174314687414272 | Second | Term | 859333475903705088 | Term | 31 |
| 1091022456318509058 | Third | Term | 788394563811540994 | Term | 32 |
| 20998237 | Second | Term | 750774917621252096 | Term | 32 |
| 2392218633 | Second | Term | 755540318959788032 | Term | 32 |
| 763395694744907776 | First | Term | 938111144182427649 | Term | 32 |
| 1252321417321943041 | Second | Term | 3248176555 | Term | 32 |
| 1389025806693801987 | Third | Term | 4484411002 | Term | 32 |
| 1465772068667068420 | First | Preterm | 4444856237 | Term | 32 |
| 462028468 | Third | Preterm | 1013873505803362305 | Term | 33 |
| 18710218 | Second | Term | 870720741389606916 | Term | 33 |
| 25867579 | Third | Term | 2888180034 | Term | 33 |
| 52945686 | First | Term | 2871007224 | Term | 33 |
| 204051090 | Second | Term | 4832613750 | Term | 33 |
| 333913059 | First | Term | 738057646318592000 | Term | 33 |
| 2654374109 | Second | Term | 733065615313895424 | Term | 33 |
| 3055671964 | Second | Term | 3873064469 | Term | 33 |
| 1002561360373342208 | Second | Term | 3438854500 | Term | 33 |
| 1271528906697342978 | Second | Term | 3388425339 | Term | 33 |
| 38855452 | Third | Term | 767387759355031553 | Term | 34 |
| 43313992 | Second | Term | 996866692209770496 | Term | 34 |
| 1023943381 | Second | Preterm | 770973056407736320 | Term | 34 |
| 23341707 | First | Term | 961897964154302464 | Term | 35 |
| 181056650 | Second | Term | 2717454373 | Term | 35 |
| 1061986729 | Second | Term | 913553517964677121 | Preterm | 35 |
| 1343264246 | Second | Term | 905920085545635840 | Preterm | 35 |
| 1260196407505543168 | Third | Term | 2519606465 | Term | 35 |
| 15335662 | Second | Term | 998659426092494848 | Term | 36 |
| 14868531 | Third | Term | 933938199256227841 | Term | 36 |
| 20950105 | First | Term | 808949423514484736 | Term | 36 |
| 1102344930 | Third | Term | 780321582308818944 | Term | 36 |
| 2169727757 | Third | Term | 751067676227895296 | Term | 36 |
| 2360676361 | First | Term | 748701547807268864 | Term | 36 |
| 2799201483 | First | Term | 823286541380370432 | Term | 36 |
| 1001579442907615232 | Third | Term | 814499430401540096 | Term | 36 |
| 41588840 | First | Term | 758844805086490624 | Term | 37 |
| 68047423 | Third | Term | 912820978970910720 | Term | 37 |
| 310085287 | Second | Term | 750563976136757251 | Term | 37 |
| 343631821 | First | Term | 930860829251133442 | Term | 37 |
| 423032117 | Second | Term | 896879222353829888 | Term | 37 |
| 21184942 | Second | Term | 1025387344533364736 | Term | 38 |
| 22896040 | Second | Term | 2578982483 | Term | 38 |
| 92099758 | Second | Term | 2158601123 | Term | 38 |
| 364328200 | Third | Term | 783011093711118336 | Term | 38 |
| 2454897821 | Second | Term | 705439903484657664 | Term | 38 |
| 40705034 | Third | Term | 3847037476 | Term | 39 |
| 17330827 | Third | Term | 4855360577 | Term | 40 |
| 1374799134 | Third | Term | 999688044159668225 | Term | 40 |
| 573808102 | Third | Term | 707699394330664960 | Term | 41 |
| 1067148274744926208 | Third | Term | 481324533 | Term | 41 |
| 2488845146 | Second | Term | 78305985 | Preterm | 41 |
| 1247102334993383424 | First | Term | 33793603 | Preterm | 41 |
| 96256541 | Second | Term | 889603303562244099 | Preterm | 42 |
| 21511998 | First | Term | 703021403100958720 | Term | 42 |
